# An Engineered Human Fc variant With Exquisite Selectivity for FcγRIIIa_V158_ Reveals That Ligation of FcγRIIIa Mediates Potent Antibody Dependent Cellular Phagocytosis With GM-CSF-Differentiated Macrophages

**DOI:** 10.3389/fimmu.2019.00562

**Published:** 2019-03-27

**Authors:** Tae Hyun Kang, Chang-Han Lee, George Delidakis, Jiwon Jung, Odile Richard-Le Goff, Jiwon Lee, Jin Eyun Kim, Wissam Charab, Pierre Bruhns, George Georgiou

**Affiliations:** ^1^Department of Chemical Engineering, University of Texas at Austin, Austin, TX, United States; ^2^Department of Biomedical Engineering, University of Texas at Austin, Austin, TX, United States; ^3^Unit of Antibodies in Therapy and Pathology, Department of Immunology, Institut Pasteur, Paris, France; ^4^INSERM, U1222, Paris, France; ^5^Center for Systems and Synthetic Biology, University of Texas at Austin, Austin, TX, United States; ^6^Department of Molecular Biosciences, University of Texas at Austin, Austin, TX, United States

**Keywords:** Fc engineering, ADCP, FcgR, macrophage, monocyte

## Abstract

IgG antibodies mediate the clearance of target cells via the engagement of Fc gamma receptors (FcγRs) on effector cells by eliciting antibody-dependent cellular cytotoxicity and phagocytosis (ADCC and ADCP, respectively). Because (i) the IgG Fc domain binds to multiple FcγRs with varying affinities; (ii) even low Fc:FcγRs affinity interactions can play a significant role when antibodies are engaged in high avidity immune complexes and (iii) most effector cells express multiple FcγRs, the clearance mechanisms that can be mediated by individual FcγR are not well-understood. Human FcγRIIIa (hFcγRIIIa; CD16a), which exists as two polymorphic variants at position 158, hFcγRIIIa_V158_ and hFcγRIIIa_F158_, is widely considered to only trigger ADCC, especially with natural killer (NK) cells as effectors. To evaluate the role of hFcγRIIIa ligation in myeloid-derived effector cells, and in particular on macrophages and monocytes which express multiple FcγRs, we engineered an aglycosylated engineered human Fc (hFc) variant, Fc3aV, which binds exclusively to hFcγRIIIa_V158_. Antibodies formatted with the Fc3aV variant bind to the hFcγRIIIa_V158_ allotype with a somewhat lower K_D_ than their wild type IgG1 counterparts, but not to any other hFcγR. The exceptional selectivity for hFcγRIIIa_V158_ was demonstrated by SPR using increased avidity, dimerized GST-fused versions of the ectodomains of hFcγRs and from the absence of binding of large immune complex (IC) to CHO cells expressing each of the hFcγRs, including notably, the FcγRIIIa_F158_ variant or the highly homologous FcγRIIIb. We show that even though monocyte-derived GM-CSF differentiated macrophages express hFcγRIIIa at substantially lower levels than the other two major activating receptors, namely hFcγRI or hFcγRIIa, Fc3aV-formatted Rituximab and Herceptin perform ADCP toward CD20- and Her2-expressing cancer cells, respectively, at a level comparable to that of the respective wild-type antibodies. We further show that hFcγRIIIa activation plays a significant role on ADCC by human peripheral monocytes. Our data highlight the utility of Fc3aV and other similarly engineered exquisitely selective, aglycosylated Fc variants toward other hFcγRs as tools for the detailed molecular understanding of hFcγR biology.

## Introduction

Antibodies regulate a variety of immune responses by engaging Fcγ receptors (FcγRs) expressed on various leukocytes such as macrophages, granulocytes, dendritic cells (DCs), natural killer (NK) cells, and B cells. Immune complexes (ICs) formed by antibodies binding to multivalent antigens such as viruses and antibody-opsonized cancer or infected cells, induce activating FcγR-mediated immunoreceptor tyrosine-based activation motif (ITAM) signaling in effector cells, thereby inducing antibody-dependent cellular cytotoxicity and phagocytosis (ADCC/P) (1, 2). The activation of immune cells upon binding to ICs is regulated by the immunoreceptor tyrosine-based inhibitory motif (ITIM) signaling of the inhibitory FcγRIIb (3, 4). The ensuing signaling processes and immunological mechanisms triggered by IC binding depend on the expression level of FcγRs on various immune cell subsets and on the binding affinity of the Fc domain to the different FcγRs (2, 5). The composition of the single glycan at N297 of the human IgG1 Fc domain is known to both affect human FcγR (hFcγR) receptor affinity and, in some instances, to trigger additional signaling pathways via binding to the lectin-like type II receptors (6).

Human FcγRIIIa (hFcγRIIIa; CD16a) has been reported to be the most potent receptor for mediating ADCC (7, 8). NK cells which are considered to be the most important contributor to ADCC with peripheral blood mononuclear cells (PBMCs) as effector cells, express only hFcγRIIIa (2). hFcγRIIIa is also expressed in intermediate (CD14^hi^/CD16^+^) and non-classical (CD14^dim^/CD16^hi^) blood monocytes which are capable of inducing ADCC primarily via TNFα secretion (9, 10). Among the four human IgG subclasses, IgG1 and IgG3 have the higher affinity for hFcγRIIIa resulting in more potent ADCC *in vitro* (5, 11–13). A single nucleotide polymorphism of hFcγRIIIa at residue 158 which can be either Val and Phe is prevalent in humans. Earlier studies revealed that lymphoma patients expressing the higher affinity hFcγRIIIa_V158_ variant show improved clinical outcomes when treated with anti-CD20 (rituximab) and anti-Her2 (trastuzumab) compared to patients homozygous for the lower affinity, hFcγRIIIa_F158_ (14–17). The finding that higher affinity to hFcγRIIIa may lead to greater therapeutic potency stimulated extensive efforts to engineer hFc domains with improved binding hFcγRIIIa via either site-directed mutagenesis or glycoengineering, the latter accomplished primarily by completely abolishing or by reducing fucosylation (18–21). hFc defucosylated antibodies have up to 50-fold enhanced affinity to hFcγRIIIa and three defucosylated antibodies, anti-CCR4 mogamulizumab, anti-IL-5Ra benralizumab, and anti-CD20 obinutuzumab, have been evaluated for multiple therapeutic indications and approved for clinical use (21, 22).

Earlier reports had suggested that the improved therapeutic efficacy of antibodies having increased hFcγRIIIa affinity is due to more effective priming and activation of NK cells (8, 23, 24). However, in more recent studies it was observed that the depletion of macrophages, which are the predominant mediators of ADCP in tissues (25), abrogated the therapeutic efficacy of anti-CD20, anti-CD30, or anti-CD40 antibodies in mouse models whereas removal of NK cells or neutrophils did not significantly affect therapeutic efficacy (26–29). These results underline the critical role of tissue-resident macrophages and ADCP in anti-tumor antibody immunotherapy. ADCP is mainly attributed to signaling via hFcγRIIa, which is expressed at high levels on macrophages (30–32). Indeed, hFc-engineered antibodies with increased hFcγRIIa binding confer more potent ADCP (29, 31). Conversely, glycoengineered antibodies do not display higher affinity toward hFcγRIIa (33), but have been shown to increase macrophage phagocytic activity (34), potentially through hFcγRIIIa signaling.

hFcγRIIIa is expressed at much lower levels than hFcγRIIa in human *in vitro* monocyte-derived GM-CSF differentiated macrophages (gmMϕ) (30–32, 35). Thus, the importance of hFcγRIIIa engagement in mediating ADCP is an overlooked issue (36–38). Delineating the role of a particular hFcγR by blocking other receptors with antibodies can be problematic for two reasons: First, *in vitro* the cross-linking of the targeted hFcγR by blocking antibodies can alter the distribution of receptors on the membrane and also the blocking antibodies may impede the accessibility of other FcγR impacting the binding of immune complexes to other hFcγRs. Secondly, the co-administration of hFcγR blocking antibodies can complicate the design and interpretation of *in vivo* experiments. To circumvent these limitations our lab has been developing engineered Fc domains that have absolute selectivity for only one Fc binding protein (39, 40). The binding of IgG antibodies to hFcγRs or to the C1q component of the complement system is critically dependent on N-linked glycan at residue N297 of the Fc (41). Loss of the glycan increases the conformational flexibility of the CH2 domain resulting in very significant, albeit not complete loss of hFcγR and C1q binding and in drastically diminished effector functions (39, 42–44). Previously, our lab has developed an Fc variant, Fc5, that binds only to the high affinity hFcγRI and not to any other human FcγR. We further demonstrated that antibodies formatted with the Fc5 domain potentiate effective tumor cell death by monocyte-derived DCs via the ligation of FcγRI (39). In this work, we report on the engineering of an aglycosylated hFc domain, Fc3aV, that has essentially absolute specificity for the hFcγRIIIa_V158_ allotype. Using antibodies equipped with the Fc3aV variant, we showed that exclusive engagement of hFcγRIIIa results in potent ADCP with gmMϕ and also established the role of this receptor on ADCC with monocytes as effectors.

## Materials and Methods

### Cells And Reagents

Burkitt's lymphoma Raji cells (ATCC® CCL-86™) and SK-BR-3 breast tumor cell lines (ATCC® HTB-30™) were obtained from American Type Culture Collection. Raji cells were cultured in complete RPMI with 10% fetal bovine serum (FBS) and SK-BR-3 cells were cultured in complete DMEM with 10% FBS. The collections of CHO cells expressing FLAG-tagged hFcγRs were described previously (14, 40, 45).

Human PBMCs and neutrophils were purified from anonymous healthy volunteers using Histopaque density gradient centrifugation (Sigma-Aldrich). Neutrophils were activated with 50 ng/mL hIFNγ for 24 h. NK cells were isolated by negative immunodensity isolation using the RosetteSep Human NK Cell Enrichment Cocktail (StemCell Technologies). Human GM-CSF differentiated macrophage cells were differentiated from CD14^+^ monocytes with 50 ng/mL GM-CSF (BioLegend®) for 7 days. Ectodomains of hFcγRI, hFcγRIIa, hFcγRIIb, and hFcγRIIIa, were produced in Expi293 cells (Invitrogen). All primers were synthesized by Integrated DNA Technologies.

### Library Screening for hFcγRIIIa-Selective hFc Variant

As reported previously (40), *E. coli* cells expressing an IgG-library (E-library) were cultured overnight at 37°C with 250 rpm shaking in Terrific Broth media (TB, Difco™) with 2% (w/v) glucose, antibiotics (35 μg/ml of chloramphenicol and 50 μg/ml kanamycin). Following overnight growth, cells were diluted 1:100 in fresh TB medium and were induced with 1 mM of isopropyl-1-thio-β-D-galactopyranoside (IPTG) and 0.2% L-arabinose. Following incubation at 25°C for 20 h, the library cells were spheroplasted. as described previously (40). Spheroplasts were labeled with 10 nM dimeric hFcγRIIIa_V158_-GST-rPE and 100 nM of unconjugated dimeric hFcγRIIb-GST and sorted using a FACSAria^TM^ flow cytometer. FACS data were analyzed with FACSDiva software. For FACS screening, clones corresponding to the top 1% fluorescent events were isolated. Sorted spheroplasts were resorted immediately after initial sorting. The hFc mutant genes in the resorted spheroplasts rescued by PCR were cloned into *Sfi*I digested pPelB-AglycoT(H)-FLAG vector, and transformed into electro-competent *E. coli* Jude-1 harboring pBAD-AglycoT(L)-His. The resulting transformants were subjected to a next round of sorting and resorting.

### Expression and Purification of IgG Antibodies and hFcγRs

IgG1 antibodies and hFcγRs proteins were produced by transient transfection of Expi293F cells (Thermo Fisher Scientific) using the pcDNA3.4 vector (Thermo Fisher Scientific). Antibodies were purified by Protein A high capacity agarose resin (Thermo Fisher Scientific) affinity chromatography. 25 × PBS was added to filtered supernatants to a 1 × concentration, and the mixture was passed twice over the column. The column was washed with 100 ml of 1x PBS to remove nonspecifically bound proteins. Three milliliters of 100 mM glycine-HCl (pH 2.7) was added to elute the bound proteins, and the elution was immediately neutralized with 1 ml of 1 M Tris (pH 8.0). Samples were buffer-exchanged into pH7.4 PBS using Amicon Ultra-4 (Millipore) spin columns with a 10 kDa cutoff. The purity of purified samples was assessed by 4–20% gradient SDS-PAGE gel (NuSep).

### ELISA and SPR Analysis

Fifty microliters of 4 μg/ml of Trastuzumab IgG1 or its hFc variants were diluted in pH7.4 phosphate buffered saline (PBS) buffer and used to coat 96 well polystyrene ELISA plate (Corning) overnight at 4°C. After blocking with 1x PBS (pH 7.4) containing 0.5% BSA for 2 h at room temperature, the plate was washed 4 times with PBS containing 0.05% Tween20 (PBST), and incubated with serially diluted dimeric GST-tagged low affinity hFcγRs and monomeric his-tagged high affinity hFcγRI at room temperature for 1 h. After washing 4 times with the PBST buffer, either 1:10,000 diluted α-GST (GE Healthcare) or α-His antibody HRP conjugate were added. After washing three times with PBST, 50 μL TMB substrate was added per well (Thermo Scientific), 50 μL of 1 M H_2_SO_4_ was added to neutralize, and the absorbance at 450 nm was recorded.

For SPR analysis, Trastuzumab IgG1 or its variants were individually immobilized on CM5 sensor chips by amine coupling, as recommended by the manufacturer (GE Healthcare). Binding experiments were performed in HBS-EP buffer (10 mM HEPES pH 7.4, 150 mM NaCl, 3.4 mM EDTA, and 0.005% P20 surfactant). Serially diluted dimeric GST-tagged low affinity hFcγRs or monomeric his-tagged high affinity hFcγRI were injected at a flow rate of 30 μl/min for 60 s with a dissociation time of 5 min. The chip was regenerated after each run by sequential injection of 50 mM glycine, pH 4.0, 50 mM glycine, pH 9.5, and 3 M NaCl for 1 min each. For each run, a bovine serum albumin (BSA)-coupled surface was used as reference channel. Equilibrium dissociation constants (K_D_) for monovalent receptor binding were determined by fitting 1:1 Langmuir model to the data using BIAevaluation 3.2 software (GE Healthcare) and the K_D_s were averaged from three independent experiments (*n* = 3 for hFcγRIIa and hFcγRIIIa, *n* = 2 for hFcγRI and hFcγRIIb). The resulting sensorgrams were fit with a 1:1 Langmuir isotherm model or equilibrium binding model for monomeric hFcγRs using Biaevaluation 3.0 software.

### Antibody Binding Activities to Cancer Cells

Raji cells or SK-BR-3 cells (10^5^ cells) were incubated with various concentration of antibody variants for 30 min at 4°C and washed with 3% BSA in PBS. Raji- or SK-BR3-bound antibody levels were detected by FITC-conjugated, F(ab′)_2_ Fragment, goat Anti-Human IgG Fc (Jackson ImmunoResearch Laboratories).

### Immune Complex Binding Activities to hFcγRs

CHO cells expressing FLAG-tagged hFcγRs were described previously (14, 40, 45). ICs were also generated by mixing 10 μg/ml of Trastuzumab or Trastuzumab-Fc3aV and 5 μg/ml of PE-conjugated F(ab′)_2_ goat anti-human IgG F(ab′)_2_ (Jackson ImmunoResearch Laboratories) for 30 min at 37°C (14, 40, 45). CHO cells expressing hFcγRs were incubated with ICs for 1 h on ice and cell-bound ICs were detected by flow cytometry on a MACSQuant (Miltenyi Biotech). Data were analyzed with Flow Cytometry Analysis Software (FlowJo). The IC-binding activities to NK cells or neutrophils were also assayed and measured using same method, but using a FACS Calibur (BD Biosciences).

### *In vitro* Cell-Based Assays

#### FCGR3A SNP Genotyping

As previously described (46, 47), *FCGR3A* genes were specifically amplified using the following primers, 5′-TCC AAA AGC CAC ACT CAA AGA CAG CGC-3′ and 5′-GAT GGT GAT GTT CAC AGT CTC T-3′ and the sequence of amplified *FCGR3A* was analyzed. *FCGR3A* SNP was also confirmed by nested PCR using following primer sets; 5′-TCC AAA AGC CAC ACT CAA AGA CAG CGC-3′ and 5′-CTC TGA AGA CAC ATT TTT ACT CCC AAA-3′ for *FCGR3A* F158, and 5′-TCC AAA AGC CAC ACT CAA AGA CAG CGC-3′ and CTC TGA AGA CAC ATT TTT ACT CCC AAC for *FCGR3A* V158.

#### ADCC Assays

SK-BR-3 or Raji cells were cultured in complete DMEM or RPMI medium as above and collected by centrifugation at 300x g for 5 min. Cells were washed in PBS and labeled with 4 μM Calcein AM (Invitrogen) in PBS at 37°C with 5% CO_2_ for 30 min. Calcein-loaded cancer cells were washed twice, resuspended in RPMI medium, and seeded into a 96-well plate at 10,000 cells/well with various concentrations of IgG variants. As previously described (40, 48), PBMCs and PMNs were isolated from healthy anonymous donors using Histopaque (Sigma-Aldrich). CD14^+^ monocytes were isolated from PBMCs by magnetic bead separation (EasySep by STEMCELL Inc.) (40). Effector cells were added into a 96-well plate at 100,000 cells/well and the plates were incubated at 37°C with 5% CO_2_ for 4 h. Released calcein AM was detected at excitation and emission wavelengths of 485 and 535 nm, respectively. The percent of tumor cell lysis was calculated according to the following formula; 100 × (E-S)/(M-S), where E is the fluorescence of experimental well, S is that in the absence of antibody (tumor cells were incubated with medium and complement alone), and M is that of tumor cells with lysis buffer (Triton X-100 at 2% v/v, SDS 1% w/v, 100 mM NaCl, and 1 mM EDTA).

#### ADCP Assays

Purified monocytes were differentiated into GM-CSF differentiated macrophages (gmMϕ) by culture for 7 days in RPMI medium containing 10% FBS and 50 ng/ml GM-CSF. Harvested gmMϕ by trypsinization were seeded into 96-well plate at 10^5^ cells/well and then cultured in RPMI medium containing 10% FBS overnight. SK-BR-3 or Raji cells, were labeled with PKH67 (Sigma-Adrich) according to the manufacturer's instructions and opsonized by serially diluted IgG variants. IgG-opsonized and PKH-67-labeled cancer cells were added into gmMϕ-attached 96-well plate at 10^4^ cells/well. After 2 h at 37°C with 5% CO_2_, gmMϕ cells were detached from the plate by Trypsin-EDTA treatment for 20 min. gmMϕ were stained with anti-CD45-APC (BD bioscience) for ADCP assay with SK-BR-3 cells on ice for 15 min. For ADCP assay with Raji cells, gmMϕ were stained with anti-CD14-APC and anti-CD11b-APC (Biolegend) on ice for 15 min. Phagocytosis was evaluated by FACSAria™ (BD Bioscience), and reported as the fraction of double positive cells over the total number of tumor cells in the sample.

Blockade assays for hFcγRIIIa were performed as follows; gmMϕ were pre-incubated with 10 μg/ml of anti-CD16 mAb 3G8 F(ab′)_2_ (Ancell) (49–51) was pre-incubated with gmMϕ for 10 min. IgG-opsonized and PKH-67-labeled cancer cells were then incubated with anti-CD16 mAb 3G8 F(ab′)_2_-coated gmMϕ in RPMI1640 medium without serum. After 2 h at 37°C, the fraction of phagocytosed tumor cells was detected as described above. For all assays, an E:T ratio of 10:1 was used.

Fluorescent images of macrophages phagocytozing cancer cells were obtained by confocal microscopy using calcein AM-loaded Raji cells or SK-BR-3 cells opsonized with 20 μg/ml of antibodies and gmMϕ at 37°C for 1 h. Approximately 1 × 10^5^ labeled cancer cells and 1 × 10^5^ macrophages were co-incubated in 1 ml of RPMI medium. Subsequently, the co-incubated cells were labeled as described above. Phagocytosis was visualized by confocal microscopy using Zeiss LSM 710/Elyra S.1.

## Results

### Directed Evolution for hFcγRIIIa-Specific Binding hFc Variant

**Figure 1:**
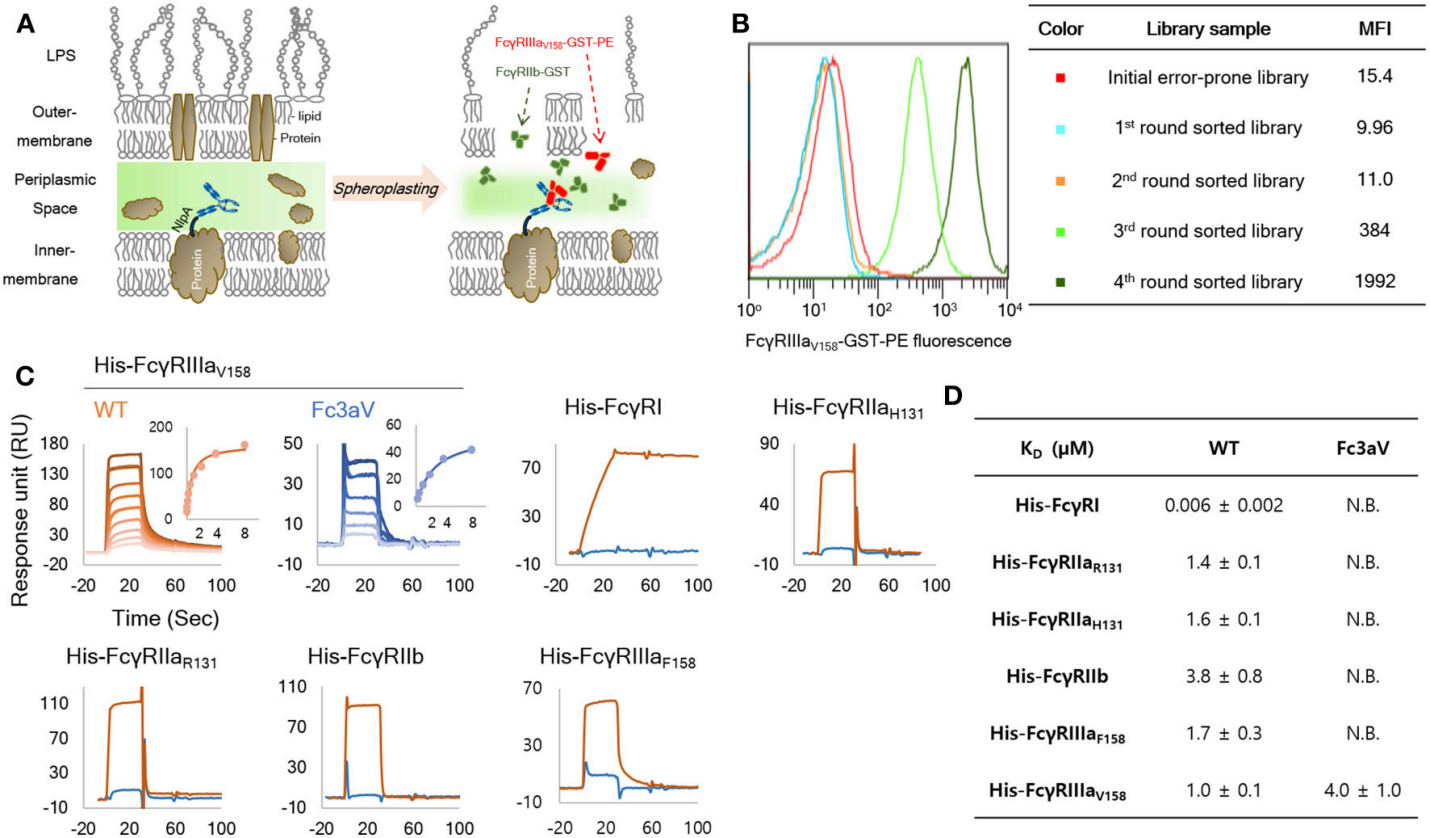
Engineering and biochemical characterization of a of FcγRIIIa_V158_-specific, Fc domain variant: **(A)** Schematic diagram of the high throughput screening system used for the isolation of FcγRIIIa_V158−_specific Fc variants from very large combinatorial libraries. Soluble fluorescent FcγRIIIa_V158_-GST and a large excess of non-fluorescent FcγRIIb-GST as a competitor are used for cell labeling and FACS sorting. **(B)** MFI (median fluorescence intensity) of initial library (red, size: 1 × 10^9^), and 1st (light blue), 2nd (orange), 3rd (light green), and 4th (green) round sorted populations. **(C,D)** SPR analysis of wild-type Trastuzumab (orange) and Trastuzumab-Fc3aV (sky blue) with monomeric His-tagged FcγRs. Surface plasmon resonance (SPR) sensorgrams **(C)** and equilibrium dissociation constants for of Fc3aV in μM **(D)**. N.B. means RUmax of sensorgram is lower than 5 RU with 8 μM of each His-hFcγR. All experiments were repeated three times and error corresponds to standard deviation (s.d.).

Aglycosylated hFc domains specific for hFcγRIIIa were engineered via the screening of combinatorial libraries expressed in *E. coli* using a well-established full-length IgG-display system in bacteria (36, 40). Briefly, the heavy chain and light chains of IgG (having Trastuzumab Fab arms) were separately expressed by fusing pelB and NlpA, respectively resulting in tethering of IgG on the periplasmic side of the inner membrane. In order to isolate hFcγRIIIA-selective aglycosylated hFc variants, the IgG hFc domain was mutagenized by error-prone PCR and a library of >10^9^ *E. coli* transformants was generated. The bacterial cells (40) were spheroplasted and the library was screened with 10 nM of dimeric hFcγRIIIA_V158_-GST, conjugated with rPE in the presence of 100 nM of unconjugated hFcγRIIB-GST as a competitor (Figures 1A,B). Ninety-six individual clones from the 4th round of sorting were randomly picked and analyzed further. The 96 clones encoded 22 unique hFc variants in which S239T, V264E, V282M, T299A, L309Q, and A378V amino acid mutations were enriched (Figure S1). Interestingly, 16 out of 22 clones contained a T299A substitution in the C'E loop of CH2 domain. The T299A mutation disrupts the canonical N-X-T/S N-linked glycosylation motif on the hFc and impairs, but not completely abolishes, hFcγR binding [(24), Figure S3]. Bacteria displaying IgGs with three different mutant Fc domains, namely, TEMA (S239T, V264E, V282M, T299A), EAQ (V264E, T299A, L309Q), and Fc3aV (S239T, P248L, V264E, V282M, T299A, L309Q, A378V), were analyzed by FACS (Figure S2). Cells expressing TEMA, EAQ and Fc3aV Fc mutated IgGs showed 16-, 48.2-, and 62.4-fold increased binding activity for hFcγRIIIa_V158_-GST dimers, respectively, compared with wild-type (wt) aglycosylated IgG1. In addition, TEMA and Fc3aV also slightly bound to hFcγRIIa or hFcγRIIb under these experimental conditions (Figure S2).

Several antibodies containing subsets of the amino acid substitutions found in TEMA, EAQ, and Fc3aV were expressed and analyzed in terms of their binding affinity and selectivity to purified monomeric, high-affinity hFcγRI-His or to GST-fused dimeric (and thus, higher avidity) versions of the low-affinity receptors, hFcγRIIa, hFcγRIIb, and hFcγRIIIa (36, 40). Three isolated Fc variants shared two mutations, V264E and T299A. First, the effect of V264E on the hFcγR-binding properties of aglycosylated Fc domain was investigated (Figure S3). Trastuzumab-V264E/T299A, showed higher hFcγRIIIa binding and decreased hFcγRIIb binding relative to the T299A variant (Figure S3). Similarly, Trastuzumab-EAQ (V264E, T299A, L309Q) showed higher affinity for hFcγRIIIa but lower hFcγRIIb binding relative to Trastuzumab-T299A/L309Q (Figure S3). Trastuzumab-Fc3aV showed selective binding to FcγRIIIa_V158_ by ELISA, but still displayed some binding toward FcγRIIIa_F158_ and FcγRIIb (Figure S3).

Due to the low sensitivity achieved by direct ELISA, we opted to measure the Fc-FcγR interactions in a highly sensitive SPR experiment. Trastuzumab-Fc3aV showed specific binding to GST-fused dimeric hFcγRIIIa_V158_ (*K*_*D*_ = 0.2 ± 0.01 μM) without any significant binding to 400 nM of GST-fused dimeric hFcγRIIa_H131/R131_, hFcγRIIb, and hFcγRIIIa_F158_ by SPR (Figure S4A and Table S1). The equilibrium dissociation constants for the binding of Trastuzumab-Fc3aV to monomeric hFcγRs were also determined by SPR. Trastuzumab-Fc3aV exhibited no detectable response for any monomeric hFcγR except for hFcγRIIIa_V158_ to which it bound with a *K*_*D*_ = 4 ± 1.0 μM (Figures 1C,D and Figure S4B,C).

**Figure 2:**
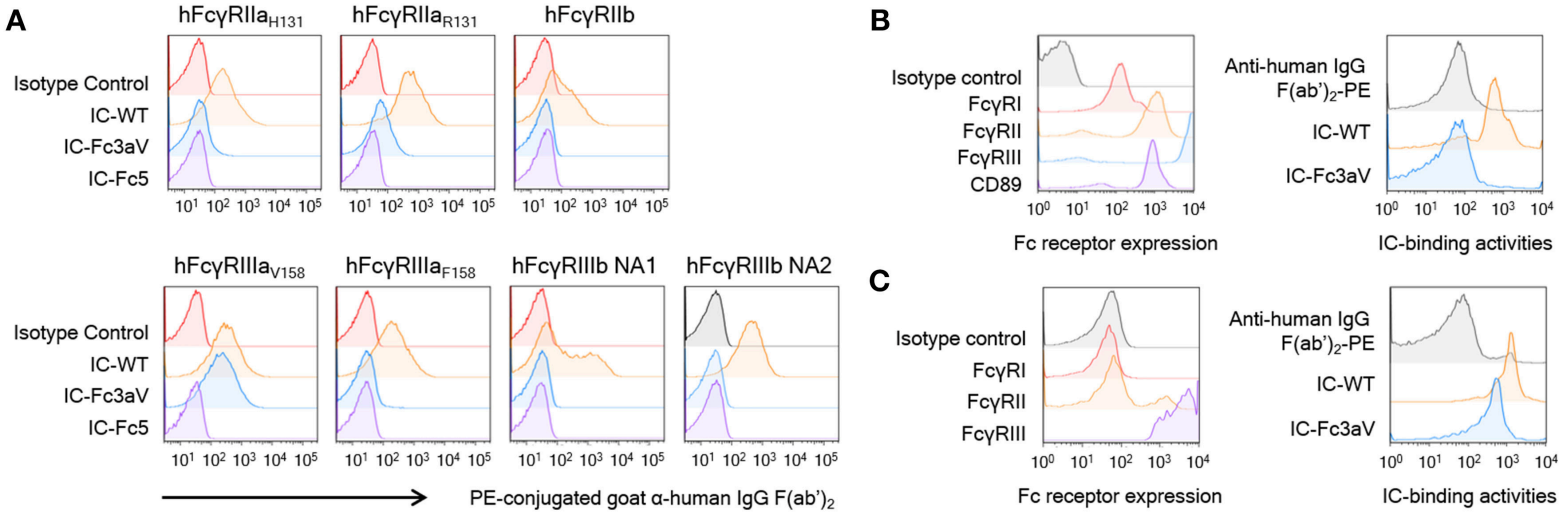
Fc3aV binds to FcγRIIIA_V158_ with exquisite selectivity **(A)** Binding activities of ICs (10 μg/ml), formed by mixing antibodies with F(ab′)_2_ anti-hIgG F(ab′)_2_-PE, to human FcγRs expressed by transfectant CHO cell measured by FACS, when antibodies are: isotype control (red), wt Trastuzumab (orange), Trastuzumab-Fc3aV (sky blue), and Trastuzumab-Fc5 (purple). **(B,C)** Binding activities of ICs on human neutrophils **(B)** or NK cells **(C)**; immune complexes were formed and used at the same concentration as in **(A)**. Left panels: Expression level of FcγRs on neutrophils activated by IFN-γ **(B)** or NK cells **(C)** detected by the respective anti-FcγR antibodies conjugated to APC; Right panels: Binding of ICs formed by wt or Fc3aV Trastuzumab, onto neutrophils or NK cells, respectively. Representative data of three independent experiments are shown.

6The binding of ICs onto cells represents an exquisitely sensitive assay for detecting physiologically relevant IgG:FcγR interactions (14, 52). Large, high avidity ICs were formed by mixing Fc3aV or other antibodies with goat F(ab′)_2_ anti-human F(ab′)_2_ and binding to CHO cells expressing the low affinity hFcγRIIa_R131_, hFcγRIIa_H131_, hFcγRIIb, hFcγRIIIa_V158_, and importantly hFcγRIIIa_F158_ which only differs from hFcγRIIIa_V158_ by one amino acid or the hFcγRIIIb-NA1 or hFcγRIIIb-NA2 allotype which differs from hFcγRIIIa by 6–8 amino acids at high levels was evaluated by FACS. IC formed by Trastuzumab Fc3aV bound to hFcγRIIIa_V158_ expressing CHO cells but did not show significant binding activities for all other hFcγRs (Figure 2A). To examine whether ICs of Trastuzumab Fc3aV bind to hFcγRI-expressing cells in a physiologically relevant context we determined binding to neutrophils from human blood, which are activated with IFN-γ. As expected, ICs formed with Trastuzumab Fc3aV did not bind to activated neutrophils which express hFcγRI, hFcγRIIa/b, and hFcγRIIIb but not hFcγRIIIa (Figure 2B) (53).

hFcγRIIIa on the surface of NK cells has a different *N*-glycan composition relative to hFcγRIIIa on the surface of monocytes/macrophages or with hFcγRIIIa ectodomain produced recombinantly in Expi293F cells. Differences in receptor glycosylation can affect the affinity for IgG (54). Nonetheless, we found that, ICs formed with Trastuzumab Fc3aV show slightly lower binding to NK cells as those formed by wild-type antibodies, a finding entirely consistent with the lower affinity of Fc3aV determined by SPR (Figures 1D,2C).

### hFcγRIIIa-Mediated Effector Functions in Various Effector Cells

**Figure 3:**
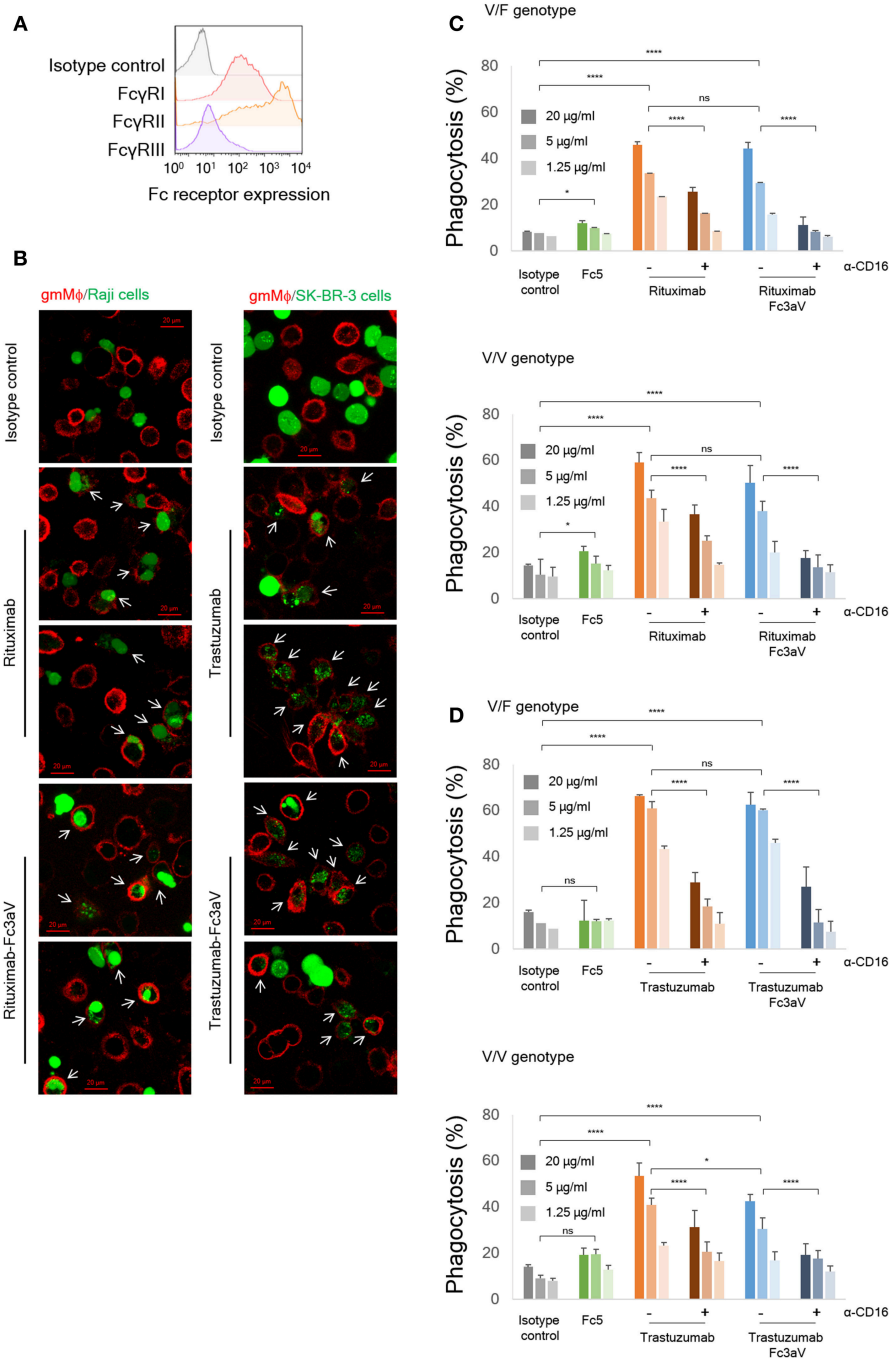
*In vitro* phagocytosis assays. **(A)** Expression levels of FcγRs on monocyte-derived human gmMϕ were detected by FACS using the respective anti-FcγR-APC antibodies. **(B)** Fluorescent images of phagocytosis of cancer cells (green, left panel: Raji and right panel: SK-BR-3) by gmMϕ (red). Macrophages were stained with anti-CD14 and anti-CD11b-APC and cancer cells were stained with Calcein-AM. White arrows indicate phagocytosed cancer cells. **(C,D)** ADCP assays with gmMϕ (V/F and V/V genotypes) as effectors and **(C)** PKH67-labeled CD20^+^ Raji cells or **(D)** PKH67-labeled Her2^high^ SK-BR-3 cells as targets (Effector:Target ratio of 10:1). Macrophages were pre-incubated with or without 10 μg/ml of anti-CD16 mAb 3G8 F(ab′)_2_ for 10 min prior to the addition of wt or variants of trastuzumab. ADCP data are shown for an FcγRIIIa heterozygous donor (V/F) and one donor homozygous for the FcγRIIIa_V158_ allotype (V/V). Representative data from for independent experiments. Error bars are standard deviations of triplicate samples. Statistical analysis was performed by two way ANOVA with Tukey's multiple comparisons test (ns: *P* > 0.05, *P ≤ 0.05, **P ≤ 0.01, ***P ≤ 0.001, ****P ≤ 0.0001).

To determine how hFcγRIIIa contributes to immune effector functions, we performed ADCP and ADCC assays using anti-CD20 Rituximab-Fc3aV or anti-Her2 Trastuzumab-Fc3aV and CD20^+^ Raji cells or Her2^high^ SK-BR-3 breast cancer cells as targets, respectively. As expected, Fc3aV-formatted antibodies with Trastuzumab or Rituximab Fab showed equivalent binding with their wt counterparts to CD20^+^ Raji cells or Her2^high^ SK-BR-3 breast cancer cells, respectively since mutations of the Fc domain did not affect antigen binding (Figure S5). We first performed ADCP assays with gmMϕ, which had been differentiated *in vitro* from human peripheral blood-derived CD14^+^ monocytes. gmMϕ express hFcγRI at moderate, hFcγRIIa/b at high and hFcγRIIIa_V158_ at low levels, as determined by flow cytometry (Figure 3A) (30–32, 35). Raji cells were labeled with fluorescent PKH67 and then opsonized with Fc-engineered Rituximab variants, followed by incubation with gmMϕ, as previously reported (40, 48). The extent of phagocytosis was evaluated using flow cytometry by determining the percentage of cells staining double positive for PKH67 and anti-CD14-APC or anti-CD11b-APC over the total number of PKH67^+^ cancer cells. We found that Rituximab-Fc3aV, which as described above binds only to hFcγRIIIa_V158_, has ADCP activity that is comparable to that of wt Rituximab (Figures 3B,C).

hFcγRIIIa-mediated phagocytosis by gmMϕ was also confirmed with Her2^high^ SK-BR-3 cancer cells opsonized with Trastuzumab-Fc3aV antibodies (Figures 3B,D). ADCP by either wt IgG- or Fc3aV- were significantly inhibited by the addition of a blocking, anti-CD16 F(ab′)_2_ [mAb clone 3G8 (49–51)], confirming the importance of hFcγRIIIa in ADCP (Figures 3C,D). Opsonization of CD20^+^ Raji cells with Rituximab equipped with Fc5, an engineered hFc domain which as discussed above engages hFcγRI selectively, resulted in a low, but statistically significant level of ADCP. However, we did not detect phagocytosis of Her2^+^ SK-BR-3 cells using Trastuzumab-Fc5 (Figures 3C,D).

**Figure 4:**
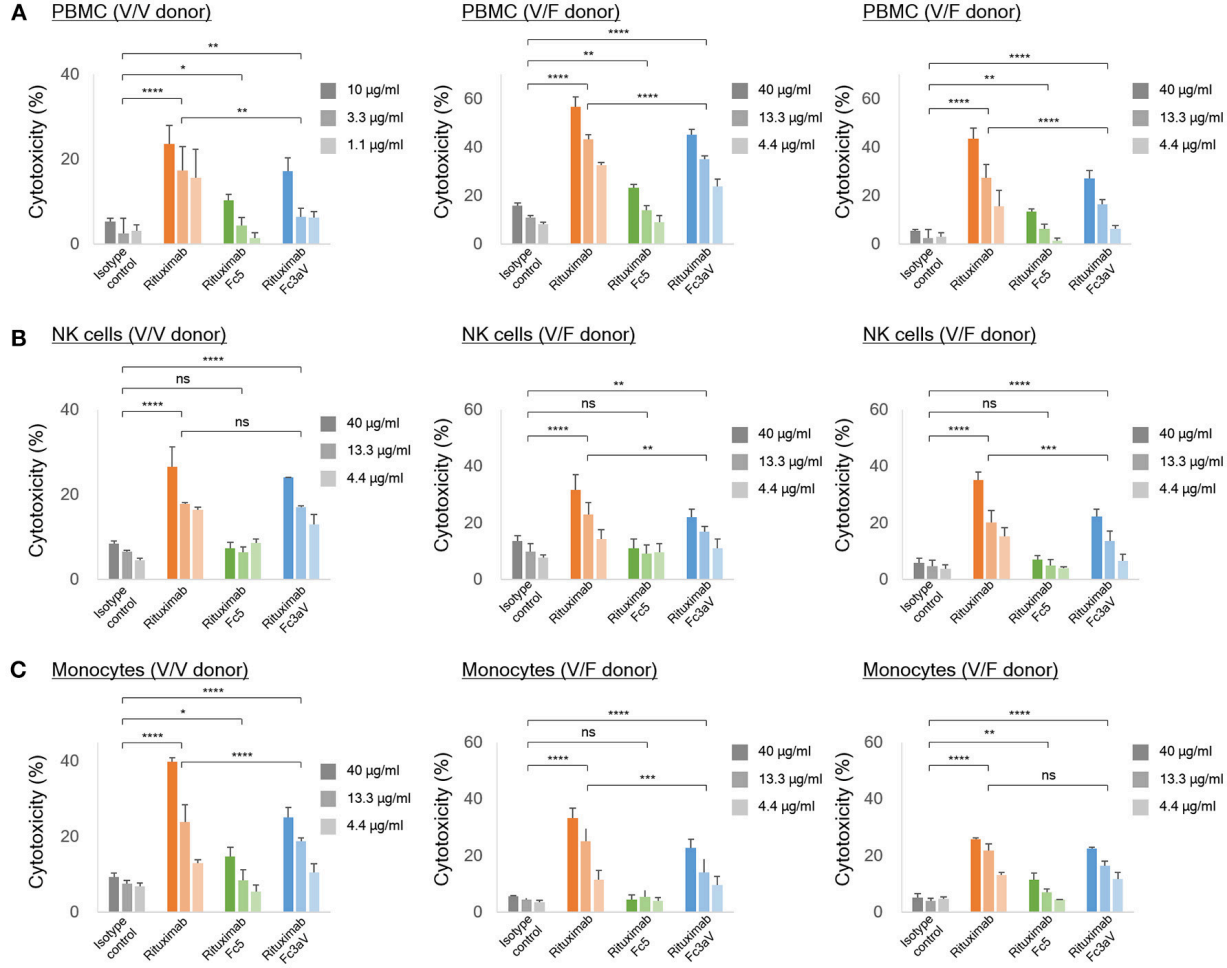
*In vitro* ADCC assays. ADCC assays with calcein-AM loaded Raji cells as targets and **(A)** PBMCs, **(B)** NK cells, and **(C)** freshly isolated monocytes from the same healthy donor [homozygous V/V (left) or heterozygous V/F (center and right) and E:T ratio = 10:1] as effector cells. **(A–C)** The level of the released calcein-AM was measured to detect target cell lysis after 4 h. Error bars correspond to standard deviations of triplicate samples. Statistical analysis was performed by two way ANOVA with Tukey's multiple comparisons test (ns: *P* > 0.05, *P ≤ 0.05, **P ≤ 0.01, ***P ≤ 0.001, ****P ≤ 0.0001).

We also determined ADCC activity by detecting calcein-AM release from cancer cells (CD20^+^ Raji cells) with PBMC, monocytes, or NK cells, isolated from the same healthy donor (V/V or V/F genotype), as effectors (*n* = 3 donors). Based on the weaker binding activity of immune complexes with Fc3aV antibodies on NK cells compared to that of w.t. IgG1 antibodies (Figure 2C), the ADCC potency of PBMCs, or NK cells using Rituximab-Fc3aV was weaker than the level observed with wt glycosylated IgG1 Rituximab (Figures 4A,B). Similarly, Rituximab-Fc3aV showed significant ADCC activity with monocytes from one V/V and two V/F donors, which was weaker than w.t. Rituximab (Figure 4C). Consistent with our findings, recently Yeap et al. presented evidence that hFcγRIIIa (CD16a) ligation is important for ADCC by monocytes. We also examined whether antibodies formatted with the hFcγRI-specific Fc5 can trigger ADCC. Rituximab-Fc5 initiated a low but statistically significant activity of ADCC with PBMCs as effectors in all donors analyzed (Figure 4A). With monocytes we only observed a low level of hFcγRI mediated ADCC in two out of three donors, consistent with the high degree of hFcγRI variability from donor to donor (Figure 4C). Finally, as expected, Fc5 formatted antibodies did not trigger ADCC with NK cells which do not express any hFcγRI (Figure 4B).

## Discussion

A number of engineered Fc domains including the SDIE (S239D/I332E), SDALIE (S239D/A330L/I332E), S298A/E333A/K334A, GASDALIE (G236A/S239D/A330L/I332E), and GASDIE (G236A/S239D/I332E) variants showing increased hFcγRIIIa affinity had been engineered (5, 55). However, all these Fcs show detectable and, in some instances, high affinity to other hFcγR and therefore they are not suitable for mechanistic studies to selectively delineate the phenotypes induced solely via the engagement of FcγRIIIa. Removal of the single glycan in the IgG hFc domain markedly increases the flexibility of the CH2 conformation resulting in dramatically decreased binding to all hFcγRs as well as to C1q (44). We had shown earlier that mutations in aglycosylated human IgG Fc domains can stabilize a particular CH2 conformer and restore binding selectively either to a single Fc binding protein, e.g., hFcγRI or C1q or, alternatively to multiple hFcγRs (36, 39, 40). Extending these earlier studies, we have now engineered an aglycosylated hFc variant, Fc3aV, with high selectivity for hFcγRIIIa_V158_ and containing seven amino acids substitutions (S239T, P248L, V264E, V282M, T299A, L309Q, and A378V). Antibodies with the Fc3aV hFc domain do not show any binding to the high affinity receptor hFcγRI or to dimeric high avidity versions of the low affinity receptors hFcγRIIa/b by SPR. Consistent with this biophysical data, ICs formed by antibodies formatted with the Fc3aV domain did not bind significantly to CHO-cells expressing low-affinity hFcγRs except hFcγRIIIa_V158_. Since hFcγRIIIa glycoproteins expressed by HEK293 or CHO cells are exclusively modified by biantennary complex-type glycans with partial sialylation (56), and the *N*-glycan composition can in turn affect its affinity for IgG (54, 57) we also evaluated binding activity of ICs to primary cells. Furthermore, immune complexes of Fc3aV antibodies bind to NK cells which express hFcγRIIIa and not to activated neutrophils which express hFcγRI, hFcγRIIa, and hFcγRIIIb but not hFcγRIIIa (Figures 2B,C).

We used Fc3aV to clarify the contribution of hFcγRIIIa to ADCP. Data from clinical studies and animal models have underscored the significance of phagocytosis in the clearance of cancer cells (7, 25–29, 58). Importantly, the depletion of macrophages in mouse models was shown to abolish the therapeutic efficacy of anti-CD20, anti-CD30, or anti-CD40 antibodies in B-cell cancer models, in contrast to depletion of NK cells or neutrophils (26–29).

While earlier reports had suggested that ADCP is mediated primarily by binding to hFcγRIIa (37) here we present evidence showing that selective engagement of human hFcγRIIIa, is sufficient to trigger potent phagocytosis by gmMϕ. The ADCP activity we observed with CD20^+^ or Her2^+^ cancer cells as targets and gmMϕ as effectors is particularly noteworthy because, as had been reported earlier and confirmed here, cell surface expression of hFcγRIIIa on gmMϕ is significantly lower than that of hFcγRIIa and FcγRI [Figure 3A,(36–38)]. One reason why selective ligation of hFcγRIIIa by Fc3aV antibodies results in ADCP that is comparable to that observed with wt Rituximab which binds to all hFcγR on macrophages and notably the much more highly expressed hFcγRIIa is that, unlike wt antibodies Fc3aV does not bind to the inhibitory hFcγRIIb and therefore is unable to trigger ITIM phosphorylation and downstream signaling processes. hFcγRIIb ligation is known to inhibit the effector functions initiated by the engagement of the activating Fc receptors including ADCP (2). We note that human macrophages show a large degree of phenotypic variations, which depend on the tissue of origin and on culture method *in vitro* and is associated with significant differences in FcγR expression levels and especially hFcγRIIIa. For example, red pulp macrophages express significantly higher levels of FcγRIIIa relative to the other FcγRs (31). Similarly *in vitro* differentiated macrophages stimulated with M-CSF have generally higher levels of hFcγRIIIa compared to gmMϕ (59, 60). Moreover, donor to donor variation effects in ADCP assays are routinely observed. Along these lines, we note that the data in Figure 3D shows that with Trastuzumab-Fc3aV antibodies (but not when using Rituximab-Fc3aV and Raji cells as targets, Figure 3C), the absolute level of phagocytosis in an hFcγRIIIa V/F heterozygous donor was slightly higher than that observed in a different donor expressing the V/V allele. The fact that high ADCP, at a level comparable to that observed with w.t. antibodies, could be mediated with the Fc3aV variant in four donors, with different target cells (Raji or SK-BR-3) and furthermore, that phagocytosis by Fc3aV antibodies was inhibited by hFcγRIIIa-blocking antibodies support our conclusion that hFcγRIIIa engagement alone is sufficient to trigger significant ADCP by gmMϕ.?NK cells which only express hFcγRIIIa and can release cytotoxic granules are considered to be the most important contributor to ADCC *in vitro* with PBMCs as effector cells (2). As expected, opsonization of tumor cells with Fc3aV antibodies triggered potent ADCC with both PBMCs and with NK cells as effectors. Similarly we found that the selective ligation of hFcγRIIIa on monocytes strongly triggers ADCC, a finding consistent with recent reports (10). As with the ADCP results discussed above, donor to donor variability precludes a comparison of subtle differences in the absolute magnitude of ADCC among donors. For example, in Figure 4, with PBMCs but not with monocytes or NK cells as effectors, the level of ADCC in a donor that is homozygous for the expression of the high affinity 158 V allele was lower than that of the V/F heterozygous donors. This may be in part due to the fact that the level of ADCC with PBMCs as effectors depends on factors other than the hFcγRIIIa allotype including, importantly, the ratio and phenotypes of monocytes to NK cells, since cytokines or receptors expressed by monocytes can impact NK cell-mediated cytotoxicity (61).

Somewhat unexpectedly we found that antibodies with the hFcγRI-selective Fc5 domain elicited much weaker ADCP with gmMϕ opsonized with Fc3aV antibodies, even though as shown in Figure 3A FACS analysis shows a >10-fold lower signal for hFcγRIIIa on gmMϕs compared that for hFcγRI. However, it should be noted that the FACS data in Figure 3A does not reflect the absolute levels of the different receptors since the FACS data were obtained with different antibodies having different affinities for their respective hFcγR antigens. This effect is not due to poor activation of hFcγRI by Fc5 antibodies as we had established earlier that Fc5 antibodies are efficient in activating hFcγRI and initiating target cell clearance by classical dendritic cells (39). The weaker effector functions associated with ligation of hFcγRI may be due to subtle, cell-specific effects related to differences in downstream signaling following ITAM phosphorylation and the elucidation of these effects will require further investigation.

In summary, functional studies with macrophages and other myeloid-derived cells capable of clearing opsonized targets using Fc3aV formatted antibodies may prove very useful for elucidating the role of hFcγRIIIa ligation on the importance of different effector functions and leukocytes subsets for the mechanism of action of therapeutic antibodies. Myeloid cells express multiple hFcγRs and therefore when they are activated with immune complexes formed by numerous antibodies the resulting effector functions represent the integrated effect due to signaling by all the hFcγRs. The use of engineered Fc domains with absolute selectivity for only one hFcγR can be a powerful tool for elucidating the subtle signaling differences and phenotypic effects elicited by each individual Fc receptor and how these ultimately contribute to the clearance of target pathogenic cells.

## Data Availability

All datasets generated for this study are included in the manuscript and/or the Supplementary Files.

## Author Contributions

TK, C-HL, and GG conceived and designed the research. C-HL, TK, GD, JJ, OG, JL, JK, and WC performed the experiments. GG, C-HL, TK, GD, JJ, and PB analyzed the data. GG, C-HL, TK, GD, JJ, JL, and PB wrote the paper.

### Conflict of Interest Statement

The authors declare that the research was conducted in the absence of any commercial or financial relationships that could be construed as a potential conflict of interest.
