# Supplementary material for: An Engineered Human Fc variant With Exquisite Selectivity for FcγRIIIaV158 Reveals That Ligation of FcγRIIIa Mediates Potent Antibody Dependent Cellular Phagocytosis With GM-CSF-Differentiated Macrophages
Source: Front Immunol. 2019 Mar 27;10:562. doi: 10.3389/fimmu.2019.00562 (PMC6448688; doi:10.3389/fimmu.2019.00562)
Supplement: Supplementary file 1 [file Data_Sheet_1.docx]

**Supplementary material**


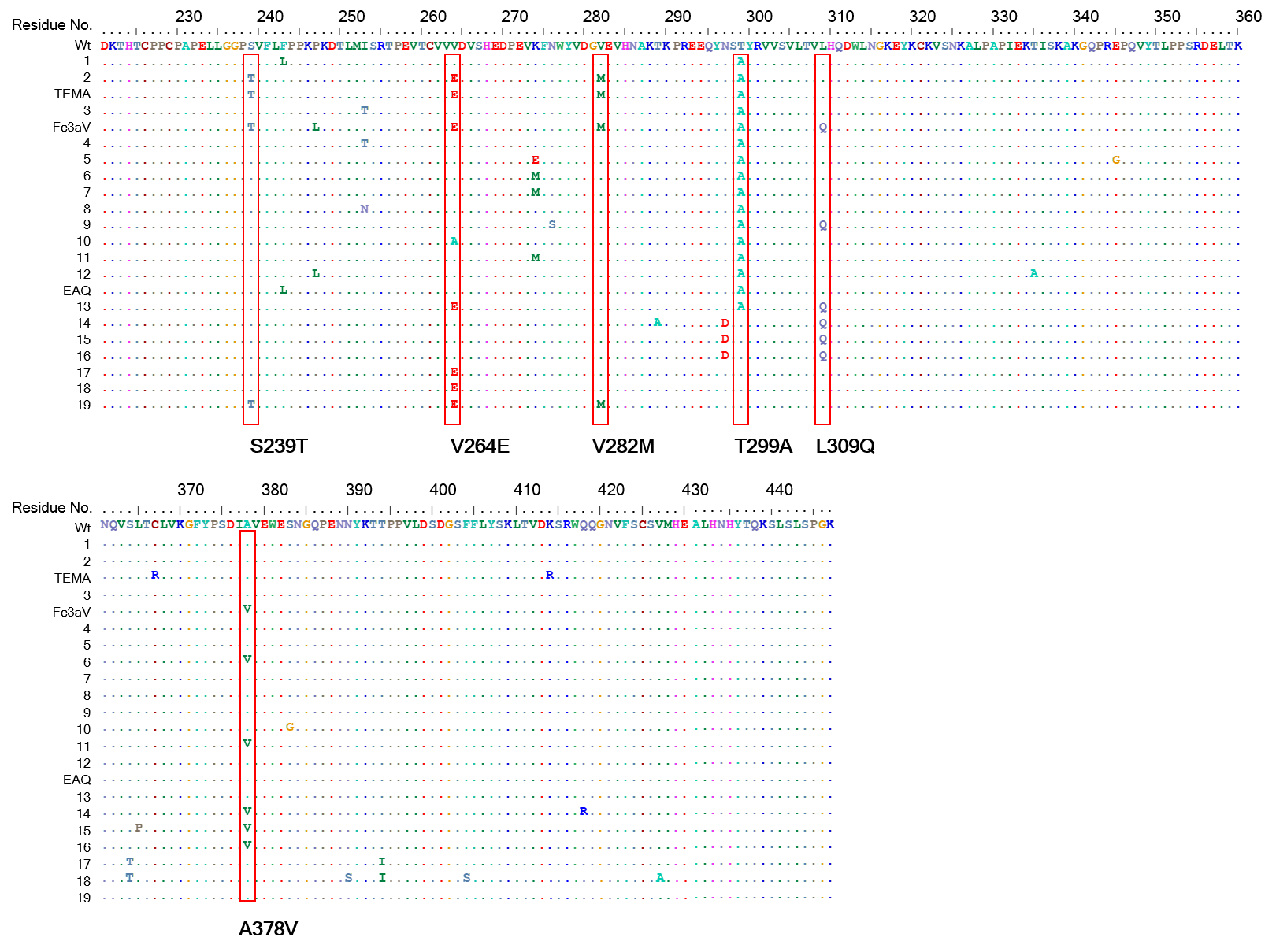


**Figure S1. Amino acids sequence alignment of isolated Fc variants.** Twenty-two Fc variants were identified from the screened library after 4 rounds of sorting. Highly enriched mutations in several Fc variants are highlighted by red box.
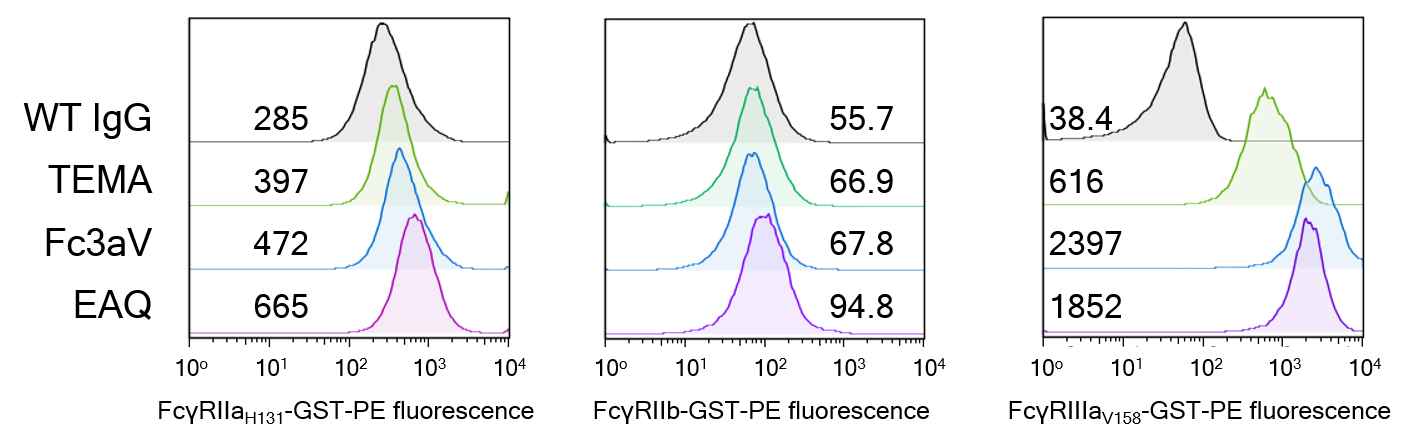


**Figure S2. Flow cytometry scanning of Fc variants isolated from library screenings.** *E.coli* spheroplasts displaying wild type, TEMA, EAQ, and Fc3aV Fc portions were incubated with 10 nM of PE-labeled GST tagged-FcγRIIa_H131_, -FcγRIIb, or -FcγRIIIa. Mean fluorescence intensity (MFI) values of GST-PE for each Fc variants are presented.

**
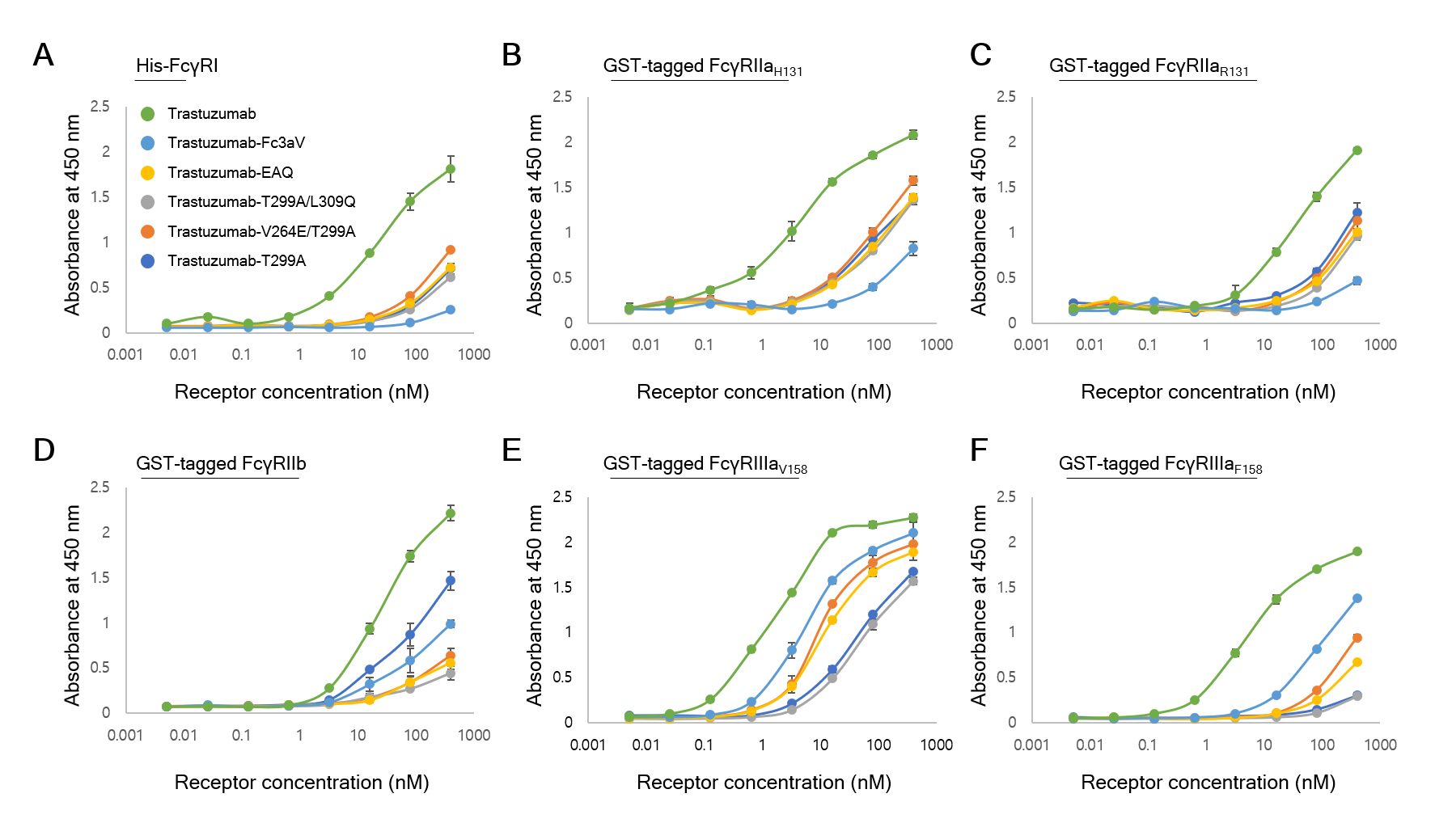
**

**Figure S3. ELISA analysis for the binding of Trastuzumab-Fc variants to ectodomains of human FcγRs.** Antibodies were coated to 96 well plates and the binding of high affinity receptor, His-tagged FcγRI (A), and low affinity receptors, GST-tagged FcγRIIa_H131_ (B), FcγRIIa_R131_ (C), FcγRIIb (D), FcγRIIIa_F158_ (E), and FcγRIIIa_V158_ (F) was detected by anti-GST antibodies coupled to HRP. Error bars represent the standard deviation from duplicate experiments.


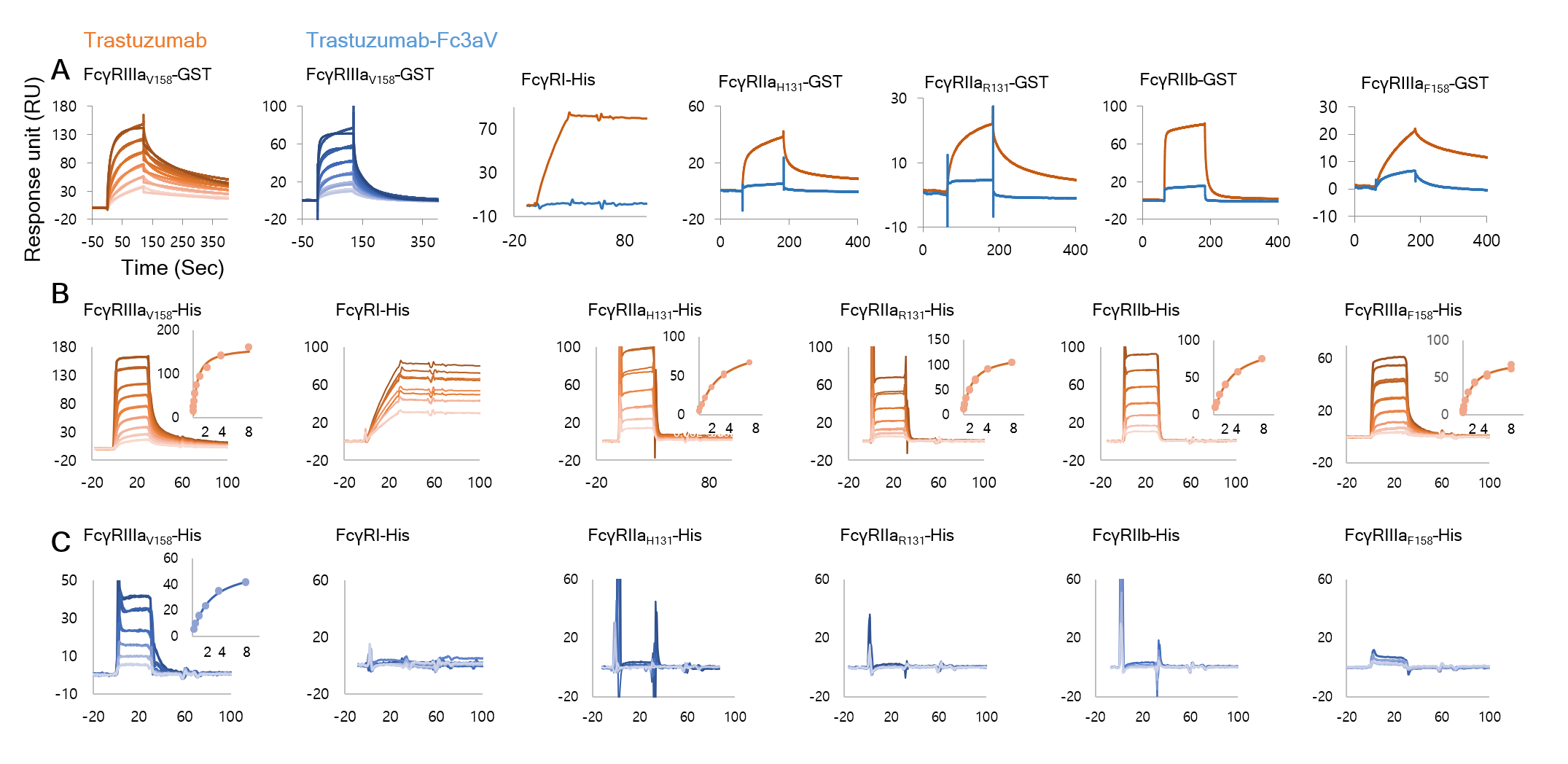


**Figure S4. Kinetic binding analysis of Trastuzumab or Trastuzumab-Fc3aV binding to FcγRs.** (**A**) SPR sensorgrams of the interaction between wt Trastuzumab (orange) or Trastuzumab-Fc3aV (sky blue) with serially diluted GST-tagged dimeric ectodomains of FcγRIIIa_V158_. Sensorgrams of the interaction between wt Trastuzumab (orange) and Trastuzumab-Fc3aV (sky blue) with 400 nM of his-tagged FcγRI or GST-tagged low-affinity FcγRs are shown using overlays. (**B-C**) SPR sensorgrams and steady state model fit of wt Trastuzumab (orange) and Trastuzumab-Fc3aV (sky blue) with either monomeric his-tagged or dimeric GST-tagged human FcγRs. Antibodies were immobilized on a CM5 chip and the binding of the serial diluted monomeric FcγRI (120 nM-40 nM), FcγRIIa_H131_, FcγRIIa_R131,_ and FcγRIIb (8 μM-125 nM) is represented. Inserted plot: Response unit (RU) as a function of receptor concentration (μM). In all sensorgrams, x axis represents time (sec) and y axis response unit (RU).


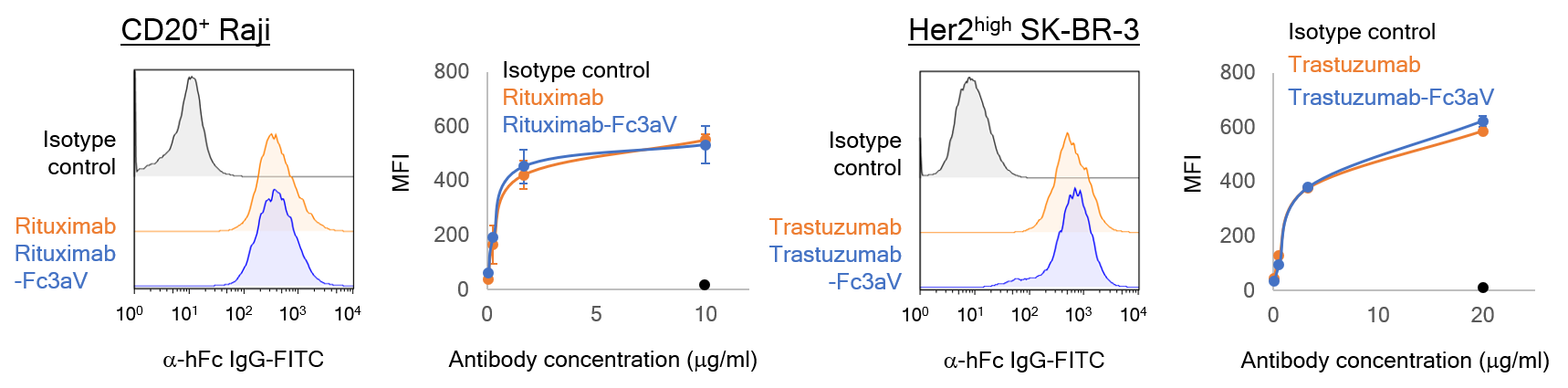


**Figure S5. Cell surface binding analysis of antibody variants with engineered Fc variant.** Binding activity of the serially diluted Rituximab or Trastuzumab Fc variant to CD20^+^ Raji or Her2^high^ SK-BR-3 cells was detected by a F(ab’)_2_ fragment against hIgG Fc, conjugated with FITC.

**Table S1. K_D_ values for the binding Fc3aV to dimeric, GST-tagged low affinity FcγRs**

| **K_D_ (µM)** | **WT** | **Fc3aV** | **T299A** | **EA** | **EAQ** | **TEMA** |
| --- | --- | --- | --- | --- | --- | --- |
| **His-FcγRI** | 0.0057 ± 0.0017 | - | 11.7 | 1.13 | 0.90 | N.T. |
| **GST-FcγRIIa_R131_** | 1.16 | - | 3.44 | 2.63 | 1.71 | N.T. |
| **GST-FcγRIIa_H131_** | 0.22 | - | 1.81 | 1.00 | 0.98 | N.T. |
| **GST-FcγRIIb** | 7.64 | - | 0.69 | 0.11 | 0.96 | - |
| **GST-FcγRIIIa_F158_** | 1.47 | - | 71.4 | 0.91 | 0.93 | - |
| **GST-FcγRIIIa_V158_** | 0.068 | 0.2 ± 0.01 | 24.9 | 0.48 | 2.60 | 1.3 ± 0.02 |

K_D_ values were calculated by fitting the experimental curves using models provided by the BIAevaluation software: the 1:1 langmuir model for ectodomains of FcγRI; the bivalent analyte model for all other ectodomains of low-affinity FcγRs. “-“ = not detectable (lower response than 5 RU at 400 nM of dimeric FcγR), N.T.= not tested.
